# Exploring Goldstein et al.’s Scalelink method of data linkage.

**DOI:** 10.23889/ijpds.v7i3.2042

**Published:** 2022-08-25

**Authors:** Mary Ann Megan Cleaton, Josie Plachta, Rachel Shipsey

**Affiliations:** 1 Office for National Statistics

## Objectives

Scalelink is an innovative probabilistic data linkage method based on correspondence analysis. Unlike the popular and widely-used Fellegi-Sunter algorithm, it does not assume linkage variable independence. It also claims to be more intuitive and computationally efficient. We aim to test this method for the first time on real-world big data.

## Approach

Scalelink uses agreement states for each linkage variable and candidate pair. These are compared to determine how frequently, for all candidate pairs, any given agreement state is held at the same time as any other agreement state (this accounts for variable dependence). The results of this comparison are inputted into a loss function and the minimisation of this function is derived within constraints to produce weights. Currently, the method is accessible via Goldstein et al.’s paper and R package. We are translating it into PySpark to enable testing on datasets that are too large to link without using distributed computing.

## Results

Initial testing of Goldstein et al.’s Scalelink method on small samples of real-world datasets shows that it performs as expected for a probabilistic linkage method, although cannot currently deal with missingness. To test the quality of the method on real-world big data, a high-quality linked dataset of the 2021 England and Wales Census and follow-up Census Coverage Survey will be used as a Gold Standard (GS). After developing a method that enables Scalelink to deal with missingness, we will apply Scalelink and automatic Fellegi-Sunter probabilistic linkage to this GS. We can thus establish and compare the precision and recall of both methods. We will also investigate linkage bias for particular demographics, test computational efficiency and estimate the clerical review burden for each method.

## Conclusion

Goldstein et al.’s Scalelink algorithm shows promise as a high quality, scalable, dependence-free linkage algorithm for use in any matching project. Here, for the first time, we research the method’s quality and feasibility with real-world big data. From this we will produce recommendations regarding its utility.

